# Nitric oxide signaling and mitophagy in Parkinson’s disease: implications for aerobic exercise-related neuroprotection

**DOI:** 10.4103/mgr.MEDGASRES-D-26-00008

**Published:** 2026-09-12

**Authors:** Yang Zhou, Yongliang Wu

**Affiliations:** 1 School of Physical Education and Health Sciences, Guangxi Science & Technology Normal University, Laibin, Guangxi Zhuang Autonomous Region, China; 2 School of Physical Education, Guangxi University of Science and Technology, Liuzhou, Guangxi Zhuang Autonomous Region, China

**Keywords:** aerobic exercise, mitochondrial dysfunction, mitochondrial quality control, mitophagy, neuroprotection, nitric oxide, oxidative stress, Parkinson’s disease, PINK1/Parkin pathway, S-nitrosylation

## Abstract

Parkinson’s disease is strongly associated with mitochondrial dysfunction and impaired mitochondrial quality control, including defective mitophagy. Aerobic exercise is increasingly recognized as a safe and accessible intervention that can improve motor and non-motor outcomes in Parkinson’s disease and may also engage mechanisms relevant to disease modification. In this review, we propose a context-dependent framework in which aerobic exercise reshapes nitric oxide signaling toward a more adaptive profile, characterized by relatively moderate, transient, and spatially restricted nitric oxide bioactivity, and we discuss how this shift may influence mitochondrial biogenesis, mitophagy initiation, and autophagic flux regulation. Rather than treating nitric oxide as uniformly protective or deleterious, we argue that its biological effects in Parkinson’s disease depend on source, concentration, duration, subcellular localization, cellular target, and surrounding redox milieu. However, direct evidence that exercise-derived nitric oxide activates these pathways in Parkinson’s disease-relevant neural tissue is still limited. We further highlight major translational gaps, including cell-type and brain-region heterogeneity, incomplete definition of exercise dose-response relationships, and the lack of validated *in vivo* biomarkers of neuronal mitophagy and nitric oxide dynamics in patients with Parkinson’s disease. Overall, aerobic exercise is a plausible modulator of mitophagy-related pathways in Parkinson’s disease, and nitric oxide is a credible contributor to this effect; however, the current evidence supports a multi-node, context-dependent model rather than a simple linear mechanism.

## Introduction

Epidemiological and clinical evidence suggest that regular aerobic exercise is a safe, accessible, and potentially beneficial non-pharmacological intervention for Parkinson’s disease (PD).^1^ It may reduce risk, slow symptom progression, and improve both motor and non-motor symptoms.^2^ Nevertheless, the mechanisms by which exercise interacts with a key pathological feature of PD, namely mitophagy-related dysfunction, remain incompletely understood. Nitric oxide (NO) is a crucial gaseous signaling molecule with context-dependent effects in the nervous system. In the nervous system, the biological consequences of NO are highly context-dependent. They are shaped not only by NO synthase (NOS) isoform, but also by NO concentration, duration of exposure, subcellular localization, cellular target, and the surrounding redox environment. Under adaptive conditions, relatively moderate and spatially restricted NO signaling can support neurovascular coupling, synaptic plasticity, and mitochondrial homeostasis.^3^,^4^,^5^ By contrast, in inflammatory and high-ROS settings, excessive and sustained NO favors nitrosative damage, including peroxynitrite (ONOO^−^) formation and disruption of mitochondrial quality control.^6^,^7^ Importantly, aerobic exercise may shift NO signaling toward a more adaptive profile. Regular exercise can increase NO bioavailability and the reserve of NO metabolites (NOx), and may promote relatively moderate, transient, and spatially restricted NO signaling under physiological conditions.^8^,^9^

This raises a key question - can aerobic exercise-modulated NO signaling restore disrupted mitophagy-related networks in PD-relevant neural contexts, and if so, through which mechanisms might this contribute to neuroprotection? Evidence indicates that NO can modulate multiple stages of autophagy through the post-translational modification process of S-nitrosylation.^10^ For instance, it exerts context-dependent bidirectional regulation on the phosphatase and tensin homolog induced putative kinase 1 (PINK1)/Parkin pathway^11^ and crucially modifies major upstream regulatory factors, such as peroxisome proliferator-activated receptor gamma coactivator 1α (PGC-1α)^12^ and downstream adaptor proteins, such as p62.^13^ Nevertheless, these scattered findings have not yet been integrated into the overall physiological context of exercise intervention. How aerobic exercise-modulated NO signaling coordinates these regulatory nodes to restore autophagic homeostasis remains incompletely understood.

This narrative review aimed to summarize current evidence on how aerobic exercise-modulated NO signaling may influence mitophagy-related pathways in PD, and to clarify the potential relevance of this interaction to neuroprotection.

## Search Strategy

We primarily searched PubMed, with supplementary searches in Web of Science and Google Scholar to identify additional mechanistic studies, recent reviews, and key original articles. The literature search was conducted between March 1 and April 20, 2026, with a final update performed on April 26, 2026. The search covered articles published from January 1, 2000 to April 26, 2026, while several earlier seminal studies were also included when they were essential for explaining foundational mechanisms of NO biology, mitophagy, or oxidative/nitrosative stress.

The following search terms and their combinations were used: “Parkinson’s disease” OR “PD”; “aerobic exercise” OR “treadmill exercise” OR “voluntary running” OR “exercise training”; “nitric oxide” OR “NO” OR “nitric oxide synthase” OR “eNOS” OR “nNOS” OR “iNOS”; “S-nitrosylation” OR “nitrosative stress”; “mitophagy” OR “mitochondrial quality control” OR “PINK1” OR “Parkin” OR “p62/SQSTM1” OR “LC3” OR “BNIP3” OR “NIX/BNIP3L” OR “FUNDC1”; and “mitochondrial dysfunction” OR “oxidative stress” OR “neuroinflammation” OR “neuroprotection”. Targeted searches were also performed for PGC-1α, AMPK, mitochondrial biogenesis, autophagic flux, PD biomarkers, and exercise-related NO bioavailability when these topics were required to support specific mechanistic sections.

Articles were first screened by title and abstract. Full texts were then reviewed when they were considered relevant to at least one of the following themes: PD pathophysiology, mitochondrial dysfunction, mitophagy regulation, NO signaling, S-nitrosylation, aerobic exercise-induced neuroprotection, or translational biomarkers. We included peer-reviewed original studies, randomized or controlled clinical studies, animal and cellular mechanistic studies, systematic reviews, meta-analyses, and authoritative narrative reviews published in English. Articles were excluded if they were not related to PD or neurodegeneration, did not address NO signaling, mitophagy, mitochondrial quality control, or exercise-related mechanisms, were not available in full text, or were not published in English.

After screening and manual cross-checking of reference lists, 95 references were included in the final narrative synthesis. The selected literature was organized around five major themes: mitophagy dysfunction in PD, aerobic exercise-induced modulation of NO signaling, NO/S-nitrosylation-dependent regulation of mitophagy-related proteins, evidence from PD animal and human studies, and translational challenges for biomarker-informed exercise or NO-targeted interventions.

## The Dual Roles and Dysregulation of Mitophagy in Parkinson’s Disease

### Major pathways of mitophagy

Mitophagy is an evolutionarily conserved selective autophagy process in eukaryotic cells, responsible for identifying and clearing dysfunctional or superfluous mitochondria. It is critical for maintaining cellular energy homeostasis, regulating metabolism, and preventing apoptosis or inflammation triggered by the accumulation of damaged mitochondria.^14^ Based on the mode of initiation, mitophagy can be broadly categorized into two major forms: the canonical PINK1/Parkin-dependent pathway and receptor-mediated, PINK1/Parkin-independent pathways (**Figure 1**).

**Figure 1:**
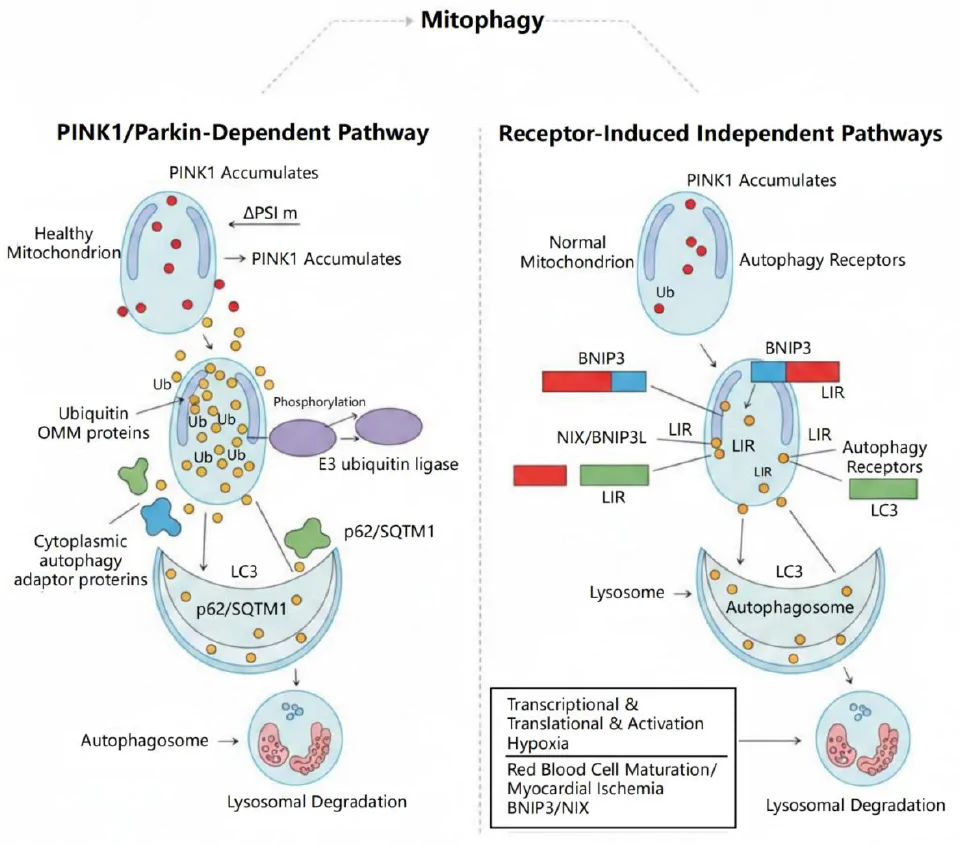
Major pathways of mitochondrial selective autophagy (mitophagy). Mitophagy proceeds through at least two major mitochondrial cargo-recognition systems. In the canonical PINK1/Parkin pathway, mitochondrial depolarization stabilizes PINK1 on the OMM, leading to phosphorylation of ubiquitin and Parkin, Parkin recruitment, OMM ubiquitination, adaptor engagement (e.g., p62/SQSTM1, OPTN), and subsequent lysosomal degradation. In receptor-mediated, PINK1/Parkin-independent mitophagy, OMM receptors such as BNIP3, NIX/BNIP3L, and FUNDC1 bind LC3/GABARAP through LIRs to recruit autophagic membranes directly. These pathways can operate independently, cooperatively, or in a compensatory manner depending on cell type and stress context. Created with Adobe Illustrator. BNIP3: Bcl2 interacting protein 3; BNIP3L: BNIP3 like; FUNDC1: Fun14 domain containing 1; GABARAP: gamma-aminobutyric acid type a receptor-associated protein; LC3: microtubule-associated protein 1 light chain 3; LIRs: LC3-interacting regions; OMM: outer mitochondrial membrane; OPTN: optineurin; PINK1: phosphatase and tensin homolog induced putative kinase 1; SQSTM1: sequestosome-1; Ub: ubiquitin.

#### PINK1/Parkin-dependent pathway

The PINK1/Parkin pathway is the best-characterized mitophagy pathway and is directly linked to the pathogenesis of familial PD.^15^ Its activation is triggered by mitochondrial depolarization, reflected by a loss of mitochondrial membrane potential (ΔΨm). In healthy mitochondria, PINK1 is imported into mitochondria, processed, and rapidly degraded. When mitochondria lose membrane potential, PINK1 import is impaired, allowing PINK1 to accumulate on the outer mitochondrial membrane (OMM), where it phosphorylates ubiquitin and promotes Parkin recruitment and activation.^16^,^17^ Accumulated PINK1 undergoes autophosphorylation and phosphorylates ubiquitin and Parkin proteins, the latter being E3 ubiquitin ligase.^18^ This phosphorylation event promotes Parkin recruitment from the cytosol to damaged mitochondria, where it ubiquitinates various OMM proteins. These ubiquitin chains serve as mitophagy signals that are recognized by selective autophagy adaptors such as p62/sequestosome-1 (SQSTM1) and optineurin (OPTN).^19^ These adaptors simultaneously bind ubiquitin chains and microtubule-associated protein 1 light chain 3 (LC3) on the autophagosomal membrane, thereby promoting the engulfment of damaged mitochondria and their subsequent lysosomal degradation.^15^,^18^ Essentially, PINK1 functions as a sensor for mitochondrial damage, while Parkin functions as an effector, jointly forming a complex ubiquitin-dependent mitochondrial quality control system.^18^

#### Receptor-mediated PINK1/Parkin-independent pathways

In addition to the PINK1/Parkin pathway, mitophagy can also be initiated through receptor-mediated, PINK1/Parkin-independent mechanisms. OMM receptors such as BNIP3, NIX/BNIP3L, and FUNDC1 contain LC3-interacting regions that directly recruit autophagic membranes.^20^,^21^,^22^ These pathways are regulated by stress-responsive signaling, including hypoxia- and phosphorylation-dependent mechanisms, and can act independently of, cooperatively with, or compensatorily relative to PINK1/Parkin.^23^,^24^ Importantly, receptor-mediated mitophagy is also functionally linked to mitochondrial dynamics, especially in the case of FUNDC1, which coordinates mitophagy with mitochondrial fission machinery under stress conditions.^21^

### Genetic evidence of mitophagy in Parkinson’s disease

Genetic studies provide some of the strongest evidence that impaired mitophagy and mitochondrial quality control are central contributors to PD pathogenesis. Among these, pathogenic mutations in PINK1 and PARK2 (which encodes Parkin), linked to autosomal recessive forms of PD, provide strong genetic evidence that disruption of the PINK1/Parkin-dependent mitophagy pathway contributes causally to neuronal degeneration.^18^,^25^

Mutations in PINK1 directly impair the initiation of mitophagy. As a key sensor of mitochondrial damage, the function of PINK1 depends on its stable accumulation on OMM during mitochondrial depolarization. Pathogenic mutations can cause decreased stability of PINK1 protein, impaired kinase activity, or mitochondrial localization failure, thereby preventing its active perception and transmission of mitochondrial damage signals.^18^ As a result, downstream Parkin recruitment and activation are impaired, preventing the establishment of ubiquitin-dependent mitophagy signaling and the timely clearance of damaged mitochondria. This blockade of mitophagic flux leads to the accumulation of dysfunctional mitochondria in neurons, resulting in sustained oxidative stress, bioenergetic failure, and ultimately the degeneration of dopaminergic neurons.^20^

Parkin mutations disturb this pathway at the effector level. As a key E3 ubiquitin ligase, Parkin mutations (such as point mutations or truncation) often cause reduced enzymatic activity or impaired substrate recognition.^26^ Even when the upstream PINK1 signal is normal, Parkin, which has lost its function, cannot effectively ubiquitinate OMM proteins in damaged mitochondria, thus rendering Parkin unable to label mitochondria for clearance and recruit autophagy processes. The decrease in Parkin function not only affects mitophagy, but may also disrupt other aspects of mitochondrial quality control, such as mitochondrial dynamics and biogenesis, thereby integratively promoting mitochondrial network disorder.^26^ Therefore, loss of function in either PINK1 or Parkin ultimately leads to a large accumulation of damaged mitochondria, and toxic mitochondrial byproducts, such as excessive reactive oxygen species (ROS), exceed the compensatory ability of cells, thereby driving neuronal death, which is the key feature of neurodegeneration in PD.^27^

Overall, the genetic evidence for PINK1- and Parkin-related familial PD strongly reveals that the autophagy pathway regulated by these two gene products plays a crucial role in the survival of dopaminergic neurons, and genetic defects in this pathway are sufficient to cause PD.

### Pathophysiological evidence of mitophagy in Parkinson’s disease

In sporadic PD, which accounts for most cases, mitophagic dysfunction is also a key pathological component,^28^,^29^ although it occurs in the absence of a clear monogenic background. Abnormal α-synuclein (α-syn) aggregation and persistent oxidative stress in the PD brain together form a vicious cycle that disrupts mitophagic flux and drives progressive neuronal degeneration.^28^,^29^

#### Toxic effects of α-synuclein aggregation

The misfolding, oligomerization, and formation of Lewy bodies of α-syn within neurons are the most prominent pathological features of PD. These pathological components, composed of α-syn, exert multiple direct toxic effects on mitochondria. They can bind and interfere with the integrity of OMM, inhibit the activity of electron transport chain complex I, causing mitochondrial dysfunction and enhanced ROS production.^30^ More importantly, α-syn aggregates actively interfere with the process of mitophagy. A study has shown that oligomers or fibrils of α-syn can inhibit the normal function of the PINK1/Parkin pathway. For example, α-syn aggregates may interfere with Parkin translocation to damaged mitochondria or impair subsequent autophagosome formation, thereby limiting the clearance of mitochondrial damage.^28^ This dual insult to the mitochondrial quality-control system both causes damage and impairs repair, leading to the sustained accumulation of damaged mitochondria, increased oxidative stress, neuroinflammation, and accelerated dopaminergic neuronal loss.

#### The vicious cycle of oxidative stress

Oxidative stress is an early and key pathophysiological feature in the progression of PD.^31^ Mitochondria are both the primary site of intracellular ROS production and the primary target of ROS attacks. In the pathological environment of PD, because of genetic defects, environmental toxins, or α-syn toxicity, mitochondria produce excessive ROS, causing oxidative damage to lipids, proteins, and DNA.^32^,^33^ This widespread oxidative damage directly impairs mitophagy itself. For instance, high levels of ROS can cause inappropriate oxidative modifications of PINK1, Parkin, and autophagy receptor proteins, resulting in their inactivation. At the same time, oxidative stress can also hinder lysosomal function, affecting autophagosome–lysosome fusion and content degradation, causing a stagnation of the entire autophagic flux regulation.^34^ As a result, mitochondria damaged by oxidative stress cannot be cleared promptly. These dysfunctional mitochondria subsequently produce more ROS, further triggering oxidative damage and inhibiting autophagy, forming a self-perpetuating vicious cycle of oxidative stress, mitochondrial damage, and autophagic dysfunction, ultimately causing neuronal energy consumption and apoptosis.^34^

## Aerobic Exercise Modulates Nitric Oxide Signaling

Given the multifaceted role of NO in maintaining neurovascular coupling and neuronal integrity, its dysregulation serves as a hallmark of neurodegenerative progression. Aerobic exercise emerges as a potent non-pharmacological intervention capable of recalibrating this homeostatic imbalance. **Figure 2** shows the differential patterns of NO signaling in aerobic exercise versus pathological states.

**Figure 2:**
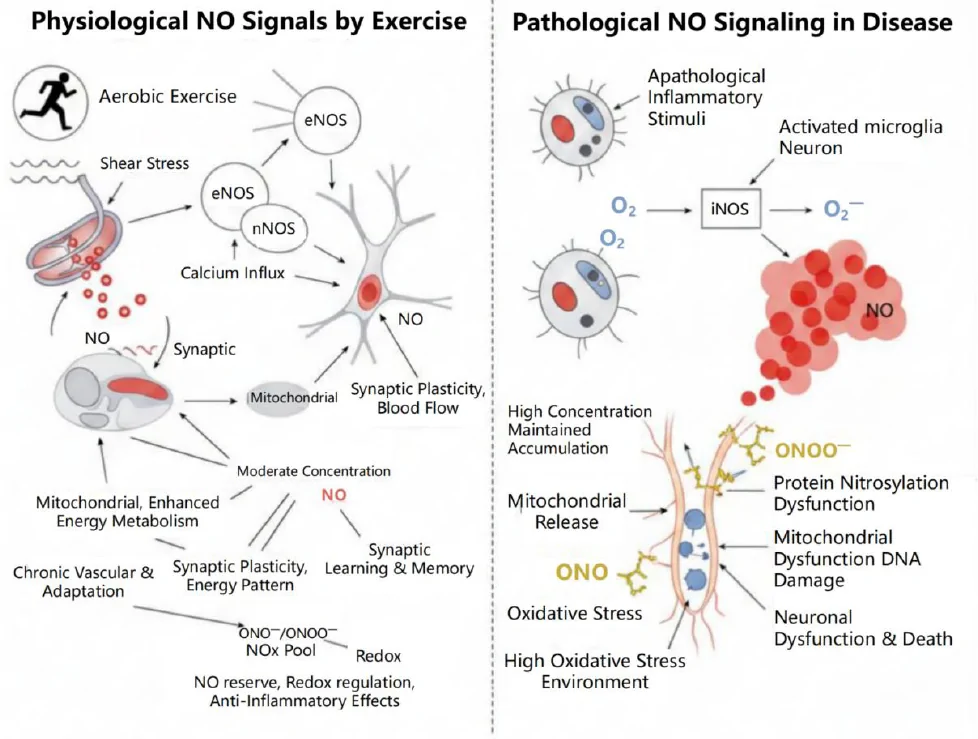
Differential mechanisms of NO signaling in aerobic exercise. During aerobic exercise, NO signaling is typically characterized by transient, spatially restricted, and moderate increases generated mainly through eNOS- and nNOS-linked pathways, together with adaptive antioxidant responses. In inflammatory or high-ROS settings, excessive and sustained NO favors peroxynitrite formation and nitrosative damage. Created with Adobe Illustrator. eNOS: Endothelial nitric oxide synthase; iNOS: inducible nitric oxide synthase; NO: nitric oxide; nNOS: neuronal nitric oxide synthase; O_2_^−^: superoxide anion; ONO^−^: nitrite; ONOO^−^: peroxynitrite; ROS: reactive oxygen species.

### Acute and chronic effects of aerobic exercise on systemic and cerebral nitric oxide homeostasis

NO is a key gaseous signaling molecule and neurotransmitter that is crucial for regulating cerebral vascular tone, neurotransmission, synaptic plasticity, and immune response.^35^ As a powerful physiological stimulus, exercise can significantly modulate NO homeostasis in a time-dependent manner in both the peripheral circulation and the central nervous system by influencing the activity and expression of different NO synthase isoforms.

The effects of exercise on NO production are source-specific and temporally dynamic. Endothelial NOS (eNOS) is a key mediator of acute vascular responses induced by exercise.^36^ During exercise, processes, such as shear stress and calcium signaling rapidly activate eNOS, causing pulsatile release of NO, which in turn causes vasodilation and increases blood perfusion to muscles and the brain. Long-term regular aerobic exercise can upregulate eNOS expression and increase its activity, promoting chronic vascular adaptation.^35^ Neuronal NOS (nNOS) is primarily enriched in neurons, notably at the postsynaptic density. Acute exercise may temporarily activate nNOS via pathways, such as calcium influx, and contribute to the regulation of neuronal excitability and synaptic function. Long-term exercise training can improve the expression of nNOS and promote long-term synaptic plasticity linked to learning and memory.^37^ In contrast, inducible NOS (iNOS) is typically induced by inflammatory stimuli and can generate high concentrations of NO that contribute to cytotoxic reactions. It has been reported that regular exercise downregulates the basal or pathological iNOS overexpression via its anti-inflammatory effect, thereby shifting NO production from a pathological pattern toward a more physiological one.^38^

The shift in NO homeostasis triggered by exercise reflects not only the immediate production of gaseous molecules, but also the stable end metabolites of nitrate (NO_3_^−^) and nitrite (NO_2_^−^; collectively referred to as NOx).^38^ A systematic review and meta-analysis involving numerous randomized controlled trials demonstrated that regular exercise training, whether aerobic or high-intensity interval training, significantly enhanced serum or plasma NO/NOx levels regardless of training duration (≤ 8 weeks or > 8 weeks).^38^ The elevation of NO-related metabolite pools has important physiological implications. It is not only a sign of enhanced NO bioavailability, but also can serve as a reserve source of NO via the nitrate–nitrite–NO pathway under hypoxic or low pH conditions, thereby continuing to modulate blood flow, mitochondrial respiration, and even autophagy processes.^39^,^40^ Nevertheless, it is worth noting that the regulation of cellular processes by NO, such as autophagy, is highly complex and concentration-dependent. *In vitro* studies have shown that high concentrations of NO, or non-physiological NO exposure, may inhibit autophagosome formation through mechanisms including S-nitrosylation.^41^ This emphasizes the important distinction between moderate, spatially and temporally limited NO signaling associated with exercise and sustained, high-level nitrosative stress typically linked to protein aggregation and autophagy flux damage in neurodegenerative diseases.^10^,^41^

Overall, aerobic exercise modulates the activity and expression of eNOS, nNOS, and iNOS in a time-dependent manner. It acutely increases local NO bioavailability to meet metabolic demands, and long-term increases steady-state NOx reserve, while improving NOS expression patterns. Together, these changes may create a molecular environment that supports energy metabolism and neuroplasticity while limiting excessive inflammation and oxidative damage.

### Exercise dose, modality, and temporal characteristics of nitric oxide-related signaling

The aerobic exercise–NO axis should not be viewed as a unitary signal. Acute exercise and chronic training differ in their biological consequences, and exercise modality, frequency, intensity, duration, and supervision level may all influence the resulting NO-related response. Acute exercise can transiently increase NO bioavailability through shear-stress- and calcium-dependent signaling, whereas chronic training may reshape baseline vascular and metabolic regulation and alter inflammatory tone.^38^,^42^ In PD, clinical studies support beneficial effects of aerobic exercise on motor outcomes and brain-related measures, but the dose-response relationship linking exercise variables to NO signaling, S-nitrosylation patterns, and mitophagy remains insufficiently defined.^42^,^43^,^44^,^45^ This gap should be acknowledged explicitly because it directly limits the mechanistic personalization of exercise prescriptions. Accordingly, individualized exercise prescriptions in PD should currently be framed as a translational goal rather than as a biomarker-validated clinical reality. A structured summary of how exercise variables may influence NO-related signaling and mitophagy-related pathways is provided in **Additional Table 1**.^36^,^38^,^42^,^44^,^46^,^47^,^48^

**Additional Table 1:** Potential effects of exercise variables on NO-related signaling and mitophagy-related pathways in PD

Exercise variable	Representative condition/example	Likely NO-related effect	Potential mitophagy relevance	Evidence strength	Key limitation	Reference
Modality	Treadmill training	May increase NO bioavailability through repeated shear stress and neural activation; may favor adaptive eNOS/nNOS-linked signaling	May support mitochondrial quality control and autophagic competence indirectly through improved redox balance and mitochondrial function	Moderate (exercise biology); limited (PD mitophagy)	Direct evidence linking treadmill-induced NO changes to neuronal mitophagy in PD is limited	36 46
Modality	Voluntary wheel running	May induce adaptive NO-related signaling with lower stress burden and distinct engagement compared with forced exercise	May engage mitophagy-related pathways in brain tissue in selected animal models	Moderate (non-PD models); limited (PD-specific)	Dose standardization and translation to human PD are limited	47
Intensity	Moderate-intensity aerobic exercise	Produces relatively moderate, transient, spatially restricted NO bioactivity within adaptive redox conditions	Compatible with preservation of mitochondrial quality control and autophagic flux regulation	Moderate (NO); indirect (mitophagy)	Lack of dose–response data linking intensity to mitophagy in PD	38,48
Intensity	High-intensity/interval exercise	May further elevate NO/NOx but also increase ROS depending on context	May enhance adaptive signaling or induce oxidative burden in vulnerable systems	Moderate (systemic NO); weak (PD mitophagy)	Threshold between beneficial and detrimental effects unclear in PD	38
Duration (acute)	Single exercise bout	Transient NO increase via shear stress and Ca^2+^ signaling	May acutely influence signaling nodes related to mitochondrial turnover	Moderate (acute NO); weak (mitophagy)	Acute responses may not translate into sustained neuronal adaptation	36
Training duration	Chronic training (weeks–months)	Reshapes baseline NO homeostasis, increases NOx reserve, improves vascular and inflammatory profile	More likely to support long-term mitochondrial quality control adaptation	Moderate (clinical relevance)	Direct longitudinal mitophagy evidence in PD lacking	38,42
Frequency	Repeated sessions	Reinforces adaptive NO signaling and redox resilience	May cumulatively support mitochondrial function and mitophagy-related processes	Indirect	Optimal frequency not defined for NO–mitophagy coupling	38
Supervision	Supervised vs. home-based exercise	Influences effective intensity and reproducibility of NO responses	Indirect via improved adherence and dose control	Moderate (clinical trials)	Mechanistic NO/mitophagy readouts rarely included	42
Disease modifiers	Disease stage, age, medication, genotype	Alters baseline NO/redox state and responsiveness to exercise	Likely modifies mitophagy-related outcomes	Conceptual strong; empirical limited	Lack of stratified mechanistic PD data	44

Ca^2+^: Calcium ion; eNOS: endothelial nitric oxide synthase; nNOS: neuronal nitric oxide synthase; NO: nitric oxide; NOx: nitrogen oxides; PD: Parkinson's disease; ROS: reactive oxygen species.

### Features of exercise-modulated nitric oxide signaling during aerobic exercise

Compared with maladaptive nitrosative signaling under inflammatory and high-ROS conditions, exercise-modulated NO signaling is better understood as a relatively moderate, transient, and spatially restricted pattern of NO bioactivity that arises within a more adaptive redox environment. These features may support vascular regulation, synaptic plasticity, mitochondrial function, and redox homeostasis, although the precise molecular consequences remain context-dependent.

#### Moderate concentration of nitric oxide

The biological effects of NO are strongly dependent on its local concentration, duration of exposure, and molecular context.^49^ Exercise-related NO is thought to arise mainly from eNOS- and nNOS-linked signaling activated by physiological stimuli such as shear stress, and is generally associated with relatively moderate and spatially restricted NO bioactivity rather than sustained high-level exposure.^48^ At moderate concentration, NO may support adaptive cellular processes, including vasodilation, synaptic plasticity, and mitochondrial signaling.^50^ By contrast, under pathological conditions such as neuroinflammation, sustained iNOS-associated NO production in a high-ROS environment is linked to nitrosative stress, growth arrest, and apoptosis.^51^ Therefore, aerobic exercise is better viewed as shifting NO signaling toward a more adaptive profile rather than as precisely maintaining NO within a single optimal concentration window.

#### Specific subcellular localization of nitric oxide

The effects of NO signaling depend not only on its concentration but also on the precise site of its production within the cell. The localization of NOSs and their product NO in various subcellular compartments indicates the spatial specificity of its bioavailability and the specific activation of downstream signaling pathways.^36^ For instance, eNOS can be localized to plasma membrane caveolae, the Golgi apparatus, or the cytoplasm, and its activity is regulated in a compartment-specific manner, resulting in distinct local NO signaling profiles.^52^,^53^ Exercise may influence the subcellular localization or compartment-specific activation of NOSs, thereby enabling NO generation at sites such as the vicinity of mitochondria or postsynaptic densities and supporting localized signaling effects.^54^,^55^

#### Pulsatile release of nitric oxide

Another key feature of physiological NO signaling is its temporal dynamics, which are pulsatile rather than constant. This release pattern is tightly linked to the physiological rhythms of exercise (e.g., heartbeat, blood flow pulsation).^56^,^57^ An *in vivo* study has shown that, compared with non-pulsatile flow, pulsatile blood flow profoundly increases both basal and stimulated release of endothelium-derived NO, indicating that pulsatility is a key physical factor modulating the kinetics of NO release.^58^ More advanced live-imaging studies have revealed that during processes, such as heart regeneration, hemodynamic forces modulate NO signaling in specific spatiotemporal patterns. This finely coordinated NO release is crucial for precisely modulating downstream regenerative responses.^59^ Accordingly, pulsatile NO release during exercise may function as a temporally encoded signal that helps modulate cellular processes requiring tight timing control, including mitochondrial quality-control pathways.

#### Cell-type and brain-region heterogeneity of mitophagy-related signaling in Parkinson’s disease

A major limitation of linear mechanistic models in PD is that mitophagy-related signaling is unlikely to be uniform across cell types or brain regions. The core lesion of PD involves dopaminergic neurons in the substantia nigra pars compacta, yet astrocytes and microglia also make substantial contributions to mitochondrial quality control, redox balance, and inflammatory signaling.^60^,^61^ Notably, PINK1-dependent ubiquitin phosphorylation has been reported to occur predominantly in astrocytes rather than neurons under some conditions, highlighting the need to avoid neuron-only interpretations of PINK1 activity.60 In parallel, glial dysfunction is increasingly recognized as a central component of PD pathogenesis, and glial iNOS-related signaling may influence the nitrosative environment experienced by neighboring neurons.^61^

Brain-region heterogeneity is also relevant. Experimental evidence suggests that mitophagy capacity differs across brain regions, with midbrain tissue appearing particularly vulnerable to defective mitochondrial turnover compared with regions showing greater compensatory reserve.^62^ Therefore, any aerobic exercise–NO–mitophagy framework for PD should be interpreted in a cell-type- and neuroanatomical region-sensitive manner rather than as a uniform whole-brain process.

### Aerobic exercise transforms nitric oxide into a physiological regulator

In the pathological progression of neurodegenerative diseases, such as PD, NO plays a complex and double-edged role. Its ultimate function, to exert neurotoxic or neuroprotective effects, is not determined by NO itself, but is regulated by the spatiotemporal patterns, concentration levels, and related redox environments produced by it.^6^,^63^ There is an important distinction between exercise-associated adaptive NO signaling and maladaptive NO signaling under pathological conditions; however, this distinction depends on signal characteristics and redox context rather than on source alone.

The source and nature of NO signals fundamentally change in the context of neuroinflammation. In pathological neuroinflammation, abnormally activated microglia and other cells can cause sustained high-level upregulation of iNOS, resulting in sustained production of large amounts of NO.^7^ Sustained high levels of NO under these conditions may contribute to neurotoxicity. More importantly, it swiftly reacts with the simultaneously produced superoxide anion (O_2_^−^) to produce the strong oxidant ONOO^−^ in a near diffusion-controlled manner.^7^,^64^ ONOO^−^ can permanently nitrify protein tyrosine residues (forming 3-nitrotyrosine), damage mitochondrial respiratory chain complexes, interfere with DNA, and directly cause neuronal dysfunction and death.^65^ By contrast, exercise-associated NO signaling is generally characterized by more moderate, transient, and spatially restricted bioactivity within a more adaptive redox environment. This signaling pattern is less likely to drive major nitrosative injury and may exert anti-inflammatory and regulatory effects by influencing immune-cell function and cytokine expression.^66^

In addition, the fate and role of NO vary greatly in high oxidative stress environments. In the pathological state of PD, intracellular O_2_^−^ levels are elevated because of factors such as mitochondrial dysfunction.^67^,^68^ Under these conditions, excess NO rapidly reacts with superoxide to form ONOO^−^, thereby shifting NO-related chemistry toward oxidative and nitrosative injury, triggering oxidative damage and forming a vicious cycle of oxidative stress and NO toxicity.^69^ Nevertheless, in physiological scenarios induced by movement, the situation is markedly different. Although exercise temporarily increases ROS production, it also upregulates the antioxidant defense system.^70^,^71^ Therefore, moderate NO release induced by exercise not only fails to trigger oxidative damage, but can also function as a significant redox regulatory signal.^70^,^71^ NO can reversibly modify mitochondrial respiratory chain enzymes or key transcription factors (e.g., via S-nitrosylation), thereby promoting mitochondrial function and improving the expression of antioxidant genes, ultimately enhancing cellular redox-buffering capacity.^48^

Overall, the distinction between maladaptive NO signaling in pathological states and adaptive NO signaling during exercise is best understood in terms of concentration, duration, localization, and redox milieu. In inflammatory settings, sustained NO production in a high-ROS environment favors ONOO^−^ formation and cellular injury. In contrast, exercise-associated NO signaling is generally more moderate and temporally restricted and may act together with adaptive antioxidant responses to support mitochondrial function and redox balance. Defining this fundamental difference is essential to elucidating how aerobic exercise counteracts the key pathology of PD by influencing more favorable NO signaling.

## Key Molecular Processes of Nitric Oxide-Induced Fine Control of Mitophagy

NO plays a vital role in regulating mitophagy.^72^ By precisely tuning various stages of mitophagy, NO can influence mitochondrial biogenesis, the clearance of damaged mitochondria, and the maintenance of homeostasis.^72^

### Modulation of upstream nitric oxide-related signaling pathways

Mitophagy does not operate in isolation but instead represents one component of the broader mitochondrial quality-control network. Its active initiation and precise execution depend on the global regulation of the mitochondrial network by upstream signals. Through S-nitrosylation and related signaling mechanisms, NO may influence upstream regulatory nodes and thereby help create conditions that are more permissive for mitophagy initiation.^73^,^74^ Among upstream regulatory nodes, PGC-1α should be discussed primarily as an NO-responsive regulator of mitochondrial biogenesis rather than as a definitively established direct S-nitrosylation target during exercise *in vivo*. Previous studies have shown that NO can regulate PGC-1α-related antioxidant and mitochondrial programs, and NO-associated signaling can cooperate with adenosine monophosphate-activated protein kinase (AMPK) to promote mitochondrial biogenesis in non-neuronal systems.^12^,^75^ At the same time, nitrosative stress may also impair the PGC-1α axis indirectly, for example, through S-nitrosylation of PARIS, a transcriptional repressor of PGC-1α, in PD-related models.^76^ Therefore, in PD, the most defensible interpretation is that exercise-modulated NO signaling may facilitate PGC-1α-related mitochondrial quality-control programs, whereas direct exercise-induced S-nitrosylation of PGC-1α in PD-relevant neural tissue remains to be demonstrated.

The relevance of the NO–PGC-1α axis lies less in demonstrating a single direct post-translational event and more in explaining how exercise-associated NO signaling may shift mitochondrial network quality toward a state that is more permissive for selective mitochondrial turnover. This interpretation is compatible with evidence linking PGC-1α to mitochondrial biogenesis, antioxidant defense, and mitochondrial quality control, but it avoids overstating the current level of *in vivo* mechanistic proof in PD (**Figure 3**).

**Figure 3:**
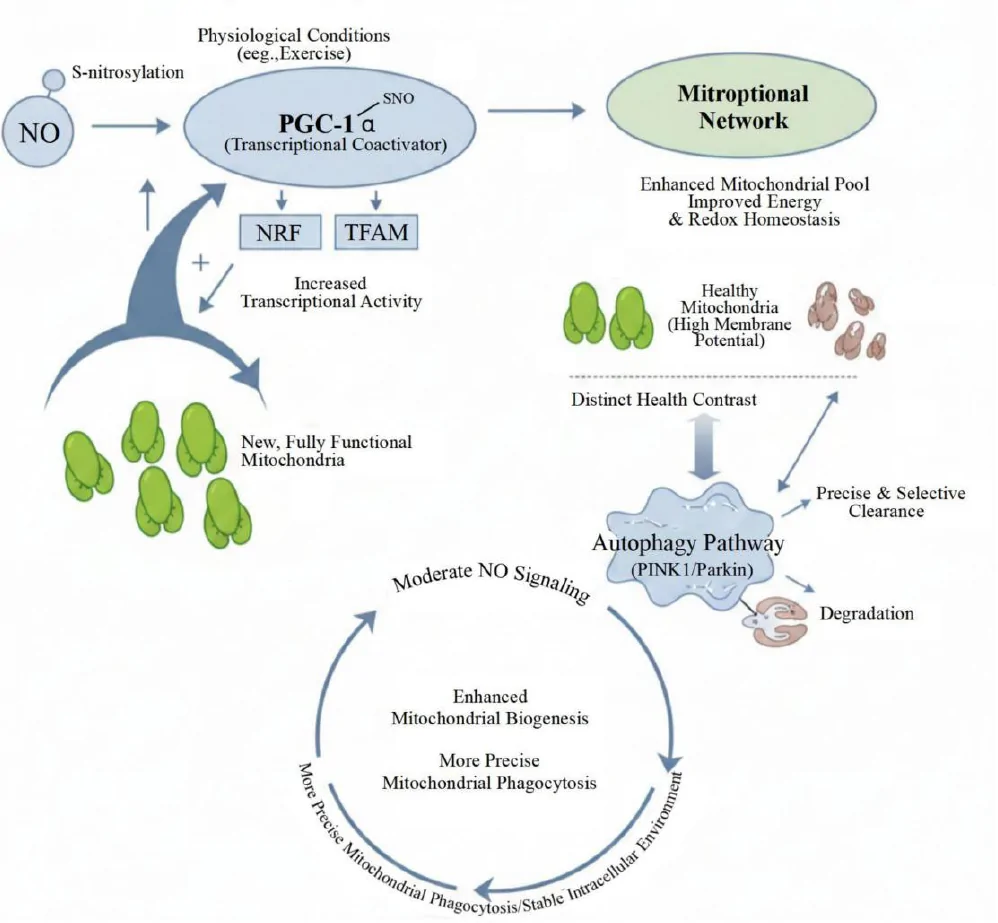
Modulation of upstream initiating signals in mitochondrial quality control. NO-associated signaling may support mitochondrial biogenesis and network remodeling through PGC-1α-related transcriptional programs, thereby improving the background quality of the mitochondrial pool. In the present framework, this upstream effect is proposed to increase the selectivity and efficiency of mitophagy by improving the contrast between healthy and dysfunctional mitochondria. Whether direct exercise-induced S-nitrosylation of PGC-1α occurs *in vivo* in PD-relevant neural tissue remains unresolved. Created with Adobe Illustrator. NO: Nitric oxide; NRF: nuclear respiratory factor; PGC-1α: peroxisome proliferator-activated receptor gamma coactivator 1α; PD: Parkinson’s disease; phosphatase and tensin homolog induced putative kinase 1; SNO: S-nitrosylation; TFAM: mitochondrial transcription factor α.

### Nitric oxide pathway modulates eat-me signals

A crucial step in mitophagy is the recognition and labeling of damaged mitochondria for clearance. This process is generally mediated by eat-me signals involving specific molecular pathways.^23^,^24^,^59^ NO may modulate this signaling by influencing the functions of numerous key molecules.^23^,^24^

#### PINK1/Parkin pathway

The PINK1/Parkin pathway is the central pathway for ubiquitin-dependent mitophagy, and its dysfunction is tightly linked to familial PD.^17^ The stable accumulation of PINK1 upon mitochondrial depolarization is the initial signal and necessary condition for activating the entire pathway.^17^ Available evidence suggests that NO-related reactive species can directly modify PINK1 through S-nitrosylation. Nevertheless, the consequence of this modification can be inhibitory. In PD models based on human induced pluripotent stem cells, S-nitrosylation of specific cysteine residues on PINK1 by NO-associated reactive nitrogen species attenuates PINK1/Parkin-dependent mitophagy.^11^ These findings suggest that, under pathological nitrosative stress, aberrant modification of PINK1 may impair its function as a mitochondrial damage sensor and thereby hinder mitophagy initiation. This highlights the potentially opposite biological effects that physiological NO signaling and pathological nitrosative stress can exert on the same target.

Parkin, as the key effector E3 ubiquitin ligase of this pathway, is regulated in a responsive and bidirectional manner by S-nitrosylation. Studies have shown that moderate S-nitrosylation can activate Parkin. Under conditions of mitochondrial depolarization, S-nitrosylation of Parkin increases its E3 ligase activity, promoting mitophagy.^77^ Nevertheless, this modulation is highly dependent on timing and concentration. Prolonged treatment with S-nitrosylating donors (e.g., GSNO) or toxins (e.g., rotenone) can cause Parkin activity to return to baseline levels.^77^ Another study has shown that under sporadic PD-associated nitrosative stress conditions, wild-type Parkin experiences S-nitrosylation and exhibits unique kinetics - initially causing a sharp increase in its E3 ubiquitin ligase activity, followed by a decline in activity, ultimately impairing the function of the ubiquitin-proteasome degradation pathway.^78^

Overall, the modulation of the PINK1/Parkin pathway by NO is significantly context-dependent. Under more adaptive conditions, moderate NO-related signaling may favor a cellular state that is more permissive for Parkin-dependent mitochondrial quality control. Conversely, sustained pathological nitrosative stress may suppress pathway initiation via abnormal modification of PINK1 or disrupt the normal activity kinetics of Parkin via excessive/aberrant modification, ultimately causing mitophagic dysfunction. This bidirectional regulatory pattern provides a useful molecular framework for understanding how exercise-associated NO signaling might alleviate nitrosative inhibition of mitophagy in PD-relevant contexts. At present, however, site-specific and dose-dependent regulation of PINK1/Parkin signaling by exercise-associated NO in PD-relevant neural tissue remains insufficiently defined.

Importantly, NO-related regulation of the PINK1/Parkin pathway is not unidirectional. Available studies suggest that nitrosative modification may impair PINK1-dependent signaling under pathological stress, whereas Parkin can exhibit biphasic or context-dependent responses to S-nitrosylation, with transient enhancement under some conditions but functional disruption under sustained nitrosative stress. The key implication is that exercise-associated NO should not be assumed to “activate” the pathway in a simple linear manner. Rather, it may shift the balance away from maladaptive nitrosative inhibition and toward a state more permissive for mitochondrial quality control.^11^,^77^,^78^

#### Receptor-mediated PINK1/Parkin-independent pathways

Compared with the PINK1/Parkin axis, evidence linking exercise-modulated NO signaling to BNIP3-, NIX-, or FUNDC1-mediated mitophagy in PD remains more indirect. These receptors are established mediators of PINK1/Parkin-independent mitophagy, and their activity is controlled by phosphorylation, mitochondrial stress, and hypoxia-responsive pathways.^21^,^22^ In addition, receptor-mediated mitophagy is functionally linked to mitochondrial dynamics, as exemplified by FUNDC1-dependent coordination of mitochondrial fission machinery.^79^ However, direct proof that exercise-derived NO activates these receptors in dopaminergic neurons is still lacking. Accordingly, these pathways are best regarded as plausible compensatory or complementary pathways rather than as established NO-driven rescue mechanisms in PD. This distinction is especially important because ULK1-dependent phosphorylation and mitochondrial dynamics can regulate receptor-mediated mitophagy independently of direct NO signaling.^21^,^79^

#### Additional mitophagy-related interacting modules

To broaden the mechanistic framework, it is important to recognize that mitophagy initiation is not limited to cargo receptors and the PINK1/Parkin module. ULK1- and ATG13-directed mitophagy has been mechanistically demonstrated,^80^ and ULK1 can phosphorylate mitophagy receptors such as FUNDC1 under stress conditions.^21^ Moreover, mitochondrial dynamics and mitophagy are tightly coupled, with fission/fusion machinery helping determine which mitochondria are segregated for clearance.^79^ In the aerobic exercise–PD context, these pathways are best viewed as interacting modules that may converge with NO-, AMPK-, and redox-sensitive signaling rather than as pathways already proven to mediate exercise-derived NO effects *in vivo*.^81^,^82^

### Nitric oxide-mediated regulation of autophagosome engulfment

NO may influence not only autophagy initiation but also key proteins involved in cargo engulfment and autophagic flux regulation through S-nitrosylation, thereby affecting the efficiency of damaged mitochondria clearance.^13^,^83^

S-nitrosylation is a key mechanism of NO signal transduction that modulates protein structure, activity, and interactions through covalent modification of cysteine residues in target proteins.^10^ The autophagic process involves the integrated action of numerous autophagy-related proteins. Among these proteins, the lipidated form of LC3 (LC3-II) is a classic marker of autophagosome membranes, while p62/SQSTM1, as a selective autophagy adaptor protein, can both recognize ubiquitin-tagged substrates (e.g., mitochondria) and bind to LC3, targeting the substrate to the forming autophagosome.^84^ NO-linked protein modification can influence autophagy at multiple stages, but the direction of the effect depends strongly on context.^41^ Under pathological nitrosative stress, excessive S-nitrosylation may suppress autophagy initiation, for example, through phosphatase and tensin homolog-related activation of protein kinase B (Akt)/mechanistic target of rapamycin (mTOR) signaling pathways,^85^ and may also impair flux at later stages, as shown for p62/SQSTM1 in α-syn-associated models.^13^ These observations argue against a simplistic view in which all NO-mediated modifications promote mitophagy. Instead, the most defensible model is that maladaptive nitrosative stress disrupts autophagic competence,^13^,^41^ whereas exercise-modulated NO signaling may help preserve or restore flux-related processes under selected conditions. The precise molecular targets mediating this beneficial effect in PD-relevant neurons remain incompletely defined (**Figure 4**).

**Figure 4:**
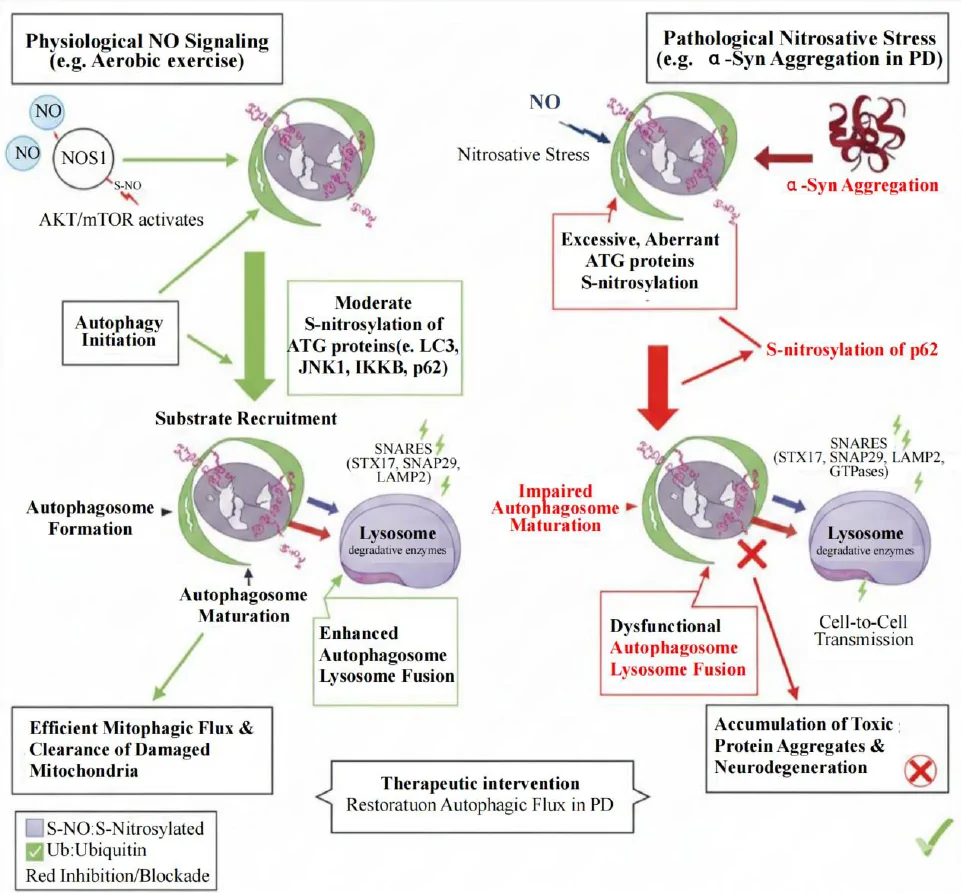
Dual role of NO-mediated S-nitrosylation in the modulation of the autophagosome engulfment phase. NO-dependent protein modification can influence autophagy initiation and flux at multiple nodes. Excessive nitrosative stress may suppress autophagy initiation, for example, through PTEN-related activation of AKT/mTOR signaling, and may also impair cargo handling or maturation, as shown for p62/SQSTM1 under α-synuclein-associated stress. By contrast, moderate exercise-related NO signaling is hypothesized to support flux competence in a context-dependent manner, although the precise molecular targets in PD-relevant neurons remain incompletely defined. Created with Adobe Illustrator. AKT: Protein kinase B; ATG: autophagy-related; IκκB: inhibitor of kappa b kinase; JNK1: c-jun N-terminal kinase 1; LAMP2: lysosomal-associated membrane protein 2; LC3: microtubule-associated protein 1 light chain 3; mTOR: mechanistic target of rapamycin; NO: nitric oxide; NOS1: nitric oxide synthase 1; PD: Parkinson’s disease; PTEN: phosphatase and tensin homolog; SNAP29: synaptosomal-associated protein 29; SNARES: soluble n-ethylmaleimide-sensitive factor attachment protein receptors; SQSTM1: sequestosome-1; STX17: syntaxin-17.

### Mitophagy as a brake on secondary oxidative and nitrosative amplification

The homeostatic value of mitophagy in PD is better understood as limiting secondary oxidative and nitrosative amplification than as directly shutting off the primary inflammatory pathway. In PD-relevant inflammatory states, iNOS is induced mainly in activated glial cells, where it contributes to a high-RNS environment.^61^,^86^ Efficient mitophagy in neurons and glia can still be protective by removing mitochondria that amplify ROS/RNS production, release mitochondrial danger-associated molecular patterns, and perpetuate redox imbalance.^86^,^87^ Thus, the resulting loop is best viewed as negative feedback on downstream mitochondrial stress propagation rather than on the upstream inflammatory trigger itself. Within this framework, aerobic exercise may support redox homeostasis not by directly eliminating inflammation, but by increasing the resilience of mitochondrial quality-control networks (**Figure 5**).

**Figure 5:**
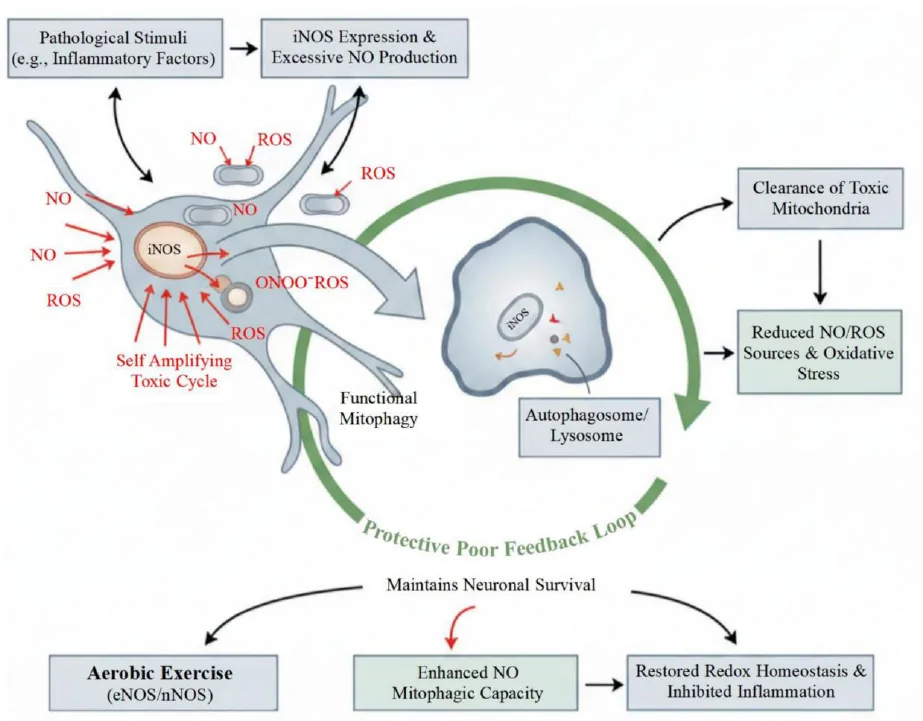
Negative feedback regulation of mitochondrial clearance and homeostatic maintenance in PD. Inflammatory activation of glia can induce iNOS and increase NO/RNS burden. Damaged mitochondria in neurons and glia can further amplify ROS/RNS production and redox imbalance. Efficient mitophagy limits this secondary amplification by removing dysfunctional mitochondria, decreasing mitochondrial danger signaling, and improving redox homeostasis. This constitutes a protective negative-feedback mechanism at the level of mitochondrial stress propagation rather than a direct shutdown of the primary inflammatory trigger. Created with Adobe Illustrator. eNOS: Endothelial nitric oxide synthase; iNOS: inducible nitric oxide synthase; nNOS: neuronal nitric oxide synthase; NO: nitric oxide; ONOO-: peroxynitrite; PD: Parkinson’s disease; RNS: reactive nitrogen species; ROS: reactive oxygen species.

## Evidence Integration, Challenges, and Prospects for Nitric Oxide-Based Therapies

**Additional Table 2** summarizes the evidence linking aerobic exercise, mitophagy, and neuroprotection across animal models and human studies.^42^,^43^,^44^,^46^,^47^,^88^,^89^,^90^,^91^,^92^

**Additional Table 2:** Evidence linking aerobic exercise, mitochondrial quality control, and translational biomarkers in PD

Evidence tier	Model/population	Intervention/design	Main finding	Mitochondrial/mitophagy-related readout	Interpretation	Reference
Direct evidence from PD animal models	MPTP-induced PD mouse model	Treadmill exercise	Improved motor deficits; reduced dopaminergic injury; reduced α-syn-related mitochondrial stress	Improved mitochondrial import machinery and mitochondrial integrity	Direct PD animal evidence for exercise-associated mitochondrial protection, but not a complete exercise–NO–mitophagy cascade	46
	MPTP-induced PD mouse model	Exercise training	Reduced neuronal apoptosis; improved mitochondrial respiration; attenuated excessive mitochondrial fission	Irisin/AMPK/SIRT1-linked mitochondrial protection	Direct PD animal evidence supporting exercise-driven mitochondrial rescue; mitophagy implication is plausible but indirect	88
Cross-disease supportive evidence for mitophagy-related mechanisms	Chronic social defeat stress mouse model	Voluntary running	Improved depressive-like behavior and neuronal ultrastructure	Activation of SIRT1–PINK1/Parkin-mediated mitophagy	Supportive mechanistic evidence, but not PD-specific	47
	APP/PS1 mouse model	Treadmill exercise	Improved mitochondrial function and cognitive outcomes	Rescue of SIRT1–FOXO1/3–PINK1/Parkin-mediated mitophagy	Supportive mechanistic evidence showing exercise can engage mitophagy-related machinery outside PD	89
Human clinical evidence	Patients with mild PD	Home-based, remotely supervised aerobic exercise RCT	Attenuated off-state motor symptom progression	No direct mitophagy readout	Clinical efficacy evidence for aerobic exercise in PD	42
	Patients with PD (RCT imaging subset)	Aerobic exercise *vs.* stretching	Altered corticostriatal functional connectivity; structural brain changes; improved cognitive control	Brain-level functional/structural correlates, but no direct mitophagy assay	Human mechanistic imaging evidence supporting brain-related exercise effects	43
	Patients with mild, early PD	6-month intense exercise program	Increased dopamine transporter availability and neuromelanin-related imaging measures	Imaging-based dopaminergic system readouts; no direct mitophagy biomarker	Translational imaging evidence consistent with neurobiological benefit	90
Human biomarker evidence and limitations	PBMCs	Functional assay after CCCP-induced mitochondrial damage	PINK1 protein undetectable; pathway not functionally engaged in PBMCs	PINK1/Parkin signaling not functionally demonstrable in PBMCs	Negative translational evidence: PBMCs are not a reliable functional surrogate of brain mitophagy	91
	Idiopathic PD plasma samples	Cross-sectional biomarker study	Plasma PINK1 elevated in PD	Plasma PINK1 as exploratory candidate biomarker	Exploratory only; not direct functional evidence of mitophagy activity	92
	Review of fluid biomarker studies in PD	Narrative/review synthesis	Exercise-responsive fluid biomarkers mainly cluster in neurotrophic, inflammatory, and neuroendocrine domains	No validated exercise-responsive mitophagy biomarker panel yet	Current translational gap: human mitophagy biomarker evidence remains insufficient	44

AMPK: Adenosine monophosphate-activated protein kinase; APP/PS1: amyloid precursor protein/presenilin 1; CCCP: carbonyl cyanide m-chlorophenyl hydrazone; FOXO1/3: Forkhead box O1/3; MPTP: 1-methyl-4-phenyl-1,2,3,6-tetrahydropyridine; NO: nitric oxide; PBMC: peripheral blood mononuclear cell; PINK1: phosphatase and tensin homolog induced putative kinase 1; RCT: randomized controlled trial; Sirt1: sirtuin 1; α-syn: α-synuclein.

### Direct evidence from Parkinson’s disease animal models

Animal studies in PD models provide the most direct support for the idea that exercise can improve mitochondrial function and neurobehavioral outcomes relevant to PD. In toxin-based models, treadmill or wheel-based exercise has been associated with improved motor performance, reduced dopaminergic neuronal loss, lower α-syn-associated pathology, and preservation of mitochondrial function or mitochondrial import machinery.^46^,^88^ However, direct measurement of NO-dependent mitophagy signaling is still limited in most exercise-PD studies. Therefore, the strongest conclusion from PD animal studies is that exercise promotes mitochondrial protection and may intersect with mitophagy-related pathways, rather than that a complete exercise–NO–mitophagy cascade has already been fully demonstrated *in vivo.*

### Cross-disease supportive evidence for mitophagy-related mechanisms

Voluntary running in chronic social defeat stress models has been linked to activation of the sirtuin 1–PINK1/Parkin axis, and treadmill exercise in APP/PS1 mice has been associated with improved mitochondrial function together with sirtuin 1–forkhead box O1/3–PINK1/Parkin signaling.^47^,^89^ These studies do not provide PD-specific evidence, but they strengthen the plausibility that exercise can engage mitophagy-related machinery across neurobiological contexts.

### Clinical and biomarker evidence

Human evidence should be considered at two levels. First, randomized and controlled studies support clinically meaningful benefits of aerobic exercise on PD-related outcomes, including attenuation of off-state motor symptom progression and exercise-associated changes in corticostriatal connectivity, brain atrophy, dopamine transporter imaging, and neuromelanin-related imaging measures.^42^,^43^,^90^ Second, direct human evidence linking these benefits to mitophagy is still limited. Available biomarker work in PD has focused mainly on neurotrophic, inflammatory, and neuroendocrine markers, whereas validated exercise-responsive mitophagy markers in blood or cerebrospinal fluid are still lacking.^44^ Plasma PINK1 and broader autophagy-lysosomal readouts should therefore be considered exploratory translational candidates rather than as definitive mechanistic biomarkers.

Exploratory biomarker studies suggest that plasma PINK1 levels may be elevated in idiopathic PD, but such findings should not be interpreted as direct functional evidence of mitophagy activity.^91^,^92^ Similarly, NO metabolites have been reported in cerebrospinal fluid or plasma in PD-related contexts, yet these measures remain heterogeneous across studies and do not currently provide a validated exercise-responsive readout of neuronal mitophagy. Peripheral biomarkers should be framed as provisional translational tools rather than definitive surrogates of brain mitophagy.^93^,^94^,^95^ Accordingly, current human studies support the clinical relevance of aerobic exercise in PD more strongly than a direct mechanistic link between exercise, NO signaling, and neuronal mitophagy.

## Current Challenges and Future Directions

Several limitations constrain the current framework. First, direct real-time measurements of neuronal NO dynamics and mitophagic flux in living patients are not yet available. Second, peripheral biomarkers of autophagy and lysosomal function remain inconsistent across biospecimens and disease subtypes, limiting their interpretability as surrogates of brain mitophagy. Third, the biological response to exercise is likely influenced by disease stage, age, sex, ancestry, medication status, and genetic background. Commonly used toxin-based animal models only partially reproduce the molecular heterogeneity of human sporadic PD. Finally, although familial and sporadic PD may converge on mitochondrial quality-control defects, their initiating lesions are not identical; therefore, mechanistic extrapolation from canonical PINK1/Parkin deficiency to sporadic PD should be made cautiously.

Future work should prioritize four directions. Multimodal biomarker studies should integrate imaging, fluid biomarkers, and digital phenotyping to capture target engagement more directly. Exercise trials in PD should standardize and report frequency, intensity, time, and type, ideally together with energy expenditure, to define biologically meaningful dose-response relationships. Mechanistic studies should resolve cell-type-specific signaling in dopaminergic neurons, astrocytes, and microglia across vulnerable and relatively spared brain regions. Fourth, pharmacological strategies designed to mimic favorable NO signaling will require substantially improved targeting and pharmacokinetic control, because conventional NO donors may suffer from short half-life, poor tissue specificity, and systemic adverse effects.^25^ Combination strategies also warrant brief but explicit consideration. Representative studies suggest that adjunctive approaches targeting redox balance, NO-related biology, or the gut-brain axis may influence PD-relevant outcomes, although they have rarely been examined alongside aerobic exercise in a unified mechanistic framework. For example, probiotic-based interventions have shown symptomatic and microbiome-associated benefits in PD, whereas antioxidant-oriented approaches and nitrate-related NO-modulating strategies have also been explored in clinical or translational contexts. In addition, preclinical evidence suggests that combining aerobic exercise with antioxidant support may improve mitochondrial damage-related outcomes in PD models. However, direct evidence that such combined strategies restore exercise-responsive, NO-dependent mitophagy in PD-relevant neural tissue is still limited. Accordingly, these approaches should currently be viewed as biologically plausible and hypothesis-generating rather than as validated therapeutic strategies.

## Conclusion

Aerobic exercise is a plausible modulator of mitophagy-related pathways in Parkinson’s disease, and NO is a biologically credible contributor to this effect. Thus, the proposed exercise–NO–mitophagy axis should currently be interpreted as a biologically plausible but incompletely validated translational framework. However, the current evidence supports a context-dependent, multi-node framework rather than a simple linear mechanism. The strongest support concerns the intersection of NO biology with mitochondrial stress signaling, the PINK1/Parkin axis, and autophagic flux regulation, whereas evidence for direct exercise-driven regulation of receptor-mediated mitophagy and for translation to human neuronal tissue remains incomplete.

## Additional files:

***Additional Table 1:*** *Potential effects of exercise variables on NO-related signaling and mitophagy-related pathways in PD.*

***Additional Table 2:*** *Evidence linking aerobic exercise, mitochondrial quality control, and translational biomarkers in PD.*

**Facts**

1. Aerobic exercise has emerged as a promising non-pharmacological strategy for Parkinson’s disease (PD).
2. Mitophagy failure is increasingly recognized as a central mechanism in PD.
3. Nitric oxide influences mitochondrial quality control in a highly context-dependent manner.
4. Exercise may reshape nitric oxide (NO) signaling toward a more adaptive biological profile.
5. The exercise–NO–mitophagy link is biologically plausible but not yet fully validated *in vivo.*

**Open Questions**

1. What is the precise dose-response relationship between aerobic exercise and NO-related mitophagy signaling in PD?
2. Which NO-sensitive molecular targets are most critical for exercise-related neuroprotection?
3. Are receptor-mediated mitophagy pathways directly engaged by exercise-derived NO in PD-relevant neural tissue?
4. How do cell-type and brain-region heterogeneity influence the exercise–NO–mitophagy axis?
5. Which translational biomarkers can reliably monitor mitophagy and NO dynamics in patients with PD?

## Data Availability

*All data generated or analyzed in this study are included in this published article and its additional files.*
